# Digital health for remote home monitoring of patients with COVID-19 requiring oxygen: a cohort study and literature review

**DOI:** 10.3389/fmed.2023.1255798

**Published:** 2024-01-31

**Authors:** Johann Chaytee, Aurélien Dinh, Emma D’Anglejan, Frédérique Bouchand, Karim Jaffal, Clara Duran, Catherine Le Gall

**Affiliations:** ^1^Emergency Department, Victor Dupouy Hospital, Argenteuil, France; ^2^Infectious Disease Department, Raymond-Poincaré Hospital, AP-HP Paris Saclay University, Garches, France; ^3^Pharmacy Department, Raymond-Poincaré Hospital, AP-HP Paris Saclay University, Garches, France

**Keywords:** COVID-19, respiratory tract infection, mortality, emergency department, oxygen therapy, telemedicine, remote monitoring

## Abstract

**Background:**

The clinical course and outcome of COVID-19 vary widely, from asymptomatic and mild to critical. Elderly patients and patients with comorbidities are at increased risk of respiratory failure and oxygen requirements. Due to the massive surge, the pandemic has created challenges for overwhelmed hospitals. Thus, the original home management of COVID-19 patients requiring oxygen and remote monitoring by a web app and a nurse at home were implemented in our center. We aimed to evaluate the outcome of patients with COVID-19 requiring oxygen who benefited from home remote monitoring management.

**Methods:**

We performed a retrospective cohort study on all COVID-19 patients requiring oxygen (< 5 L/min) who consulted from October 2020 to April 2021 at our emergency department and were managed with home remote monitoring by a web app and an in-home nurse. We also carried out a literature review of studies on COVID-19 patients requiring oxygen with remote monitoring.

**Results:**

We included 300 patients [184 (61.3%) male patients, median age 51 years]. The main comorbidities were cardiovascular disease (*n* = 117; 39.0%), diabetes mellitus (*n* = 72; 24.0%), and chronic respiratory disease (*n* = 32; 10.7%). Among the 28 (9.3%) patients readmitted to the hospital, 6 (1.9%) were hospitalized in the intensive care unit, and 3 (0.9%) died. In the multivariable analysis, risk factors for unplanned hospitalization were chronic respiratory failure (odds ratio (OR) =4.476, 95%CI 1.565–12.80), immunosuppression (OR = 3.736, 95%CI 1.208–11.552), and short delay between symptoms onset and start of telemonitoring (OR = 0.744, 95%CI 0.653–0.847). In the literature review, we identified seven other experiences of remote monitoring management. Mortality rate and unplanned hospitalization were low (maximum 1.9 and 12%, respectively).

**Conclusion:**

Our study confirms the safety of home remote monitoring of patients with COVID-19 who require oxygen, as well as our literature review. However, patients with chronic respiratory failure and immunosuppression should be closely monitored.

## 1 Introduction

In December 2019, an outbreak of pneumonia of an unknown origin occurred in Wuhan city, situated in China’s Hubei province. By January 7, 2020, Chinese scientists had identified a novel coronavirus, referred to as severe acute respiratory syndrome coronavirus 2 (SARS-CoV-2), in individuals afflicted with pneumonia caused by the virus. This condition was later designated as coronavirus disease 2019 (COVID-19) by the World Health Organization in February 2020 (1).

It quickly became evident that the virus was transmitting efficiently from person to person. Consequently, the SARS-CoV-2 infection spread rapidly from an initial cluster of cases in China to a global pandemic, leading to over 130 million confirmed cases and nearly 3 million fatalities worldwide (2, 3).

The range of clinical manifestations associated with SARS-CoV-2 infection is broad. The severity of COVID-19 varies significantly, ranging from asymptomatic or mild cases to critical and fatal outcomes (4–6). While young individuals without pre-existing health conditions tend to experience mild or no symptoms, elderly patients and those with underlying comorbidities such as cardiovascular disease, diabetes, hypertension, chronic lung issues, cancer, and kidney disease face an elevated risk of respiratory failure and the need for oxygen support.

The impact of COVID-19-associated pneumonia has strained healthcare systems globally, leading to high occupancy rates in intensive care units and exerting immense pressure on healthcare resources. A surveillance study in the United States indicated that 14% of COVID-19 patients required hospitalization, with 2% being admitted to intensive care units and a 5% mortality rate. Among hospitalized patients, an overall mortality rate of 20% was observed, primarily due to severe complications such as acute respiratory distress syndrome, septic shock, and multiorgan failure necessitating oxygen support or invasive mechanical ventilation (7–9).

The rapid global emergence of the pandemic shortly after its inception in December 2019 has underscored the need for innovative approaches to healthcare delivery that minimize disease transmission risk. Telemedicine has emerged as a secure and effective alternative to in-person consultations, enabling remote communication between patients and healthcare providers without the risk of viral exposure (2, 10–12). Teleconsultations have proven particularly valuable for suspected COVID-19 cases, facilitating remote patient assessment, monitoring, and guidance throughout the diagnostic and treatment journey (4–6).

Through telemedicine technologies, patients gain virtual access to a range of healthcare services, including medical consultations, remote patient monitoring, and prescription management. This has been especially crucial for individuals with pre-existing medical conditions who face a heightened risk of COVID-19 complications (2, 10–12).

To effectively manage the unprecedented demand on healthcare systems brought about by the COVID-19 pandemic, innovative solutions are imperative. Home-based treatment and monitoring for asymptomatic or mildly symptomatic patients can alleviate the burden on healthcare facilities while ensuring care safety and efficacy. Successful implementation requires careful patient selection, coordinated efforts, telemedicine support, technological infrastructure, workforce training, and education.

To help hospitals manage patients with COVID-19, several remote monitoring processes were set up and enabled in order to allow early discharge of patients with COVID-19 (11, 13–15). In this context, home oxygen remote monitoring could provide substantial bed savings in acute care during the pandemic.

Our hospital developed, collaboratively with primary and emergency care front-line physicians, a telemedicine solution specifically devoted to the evaluation and management of patients with COVID-19 and nasal oxygen at home to facilitate discharges. The main aspect was to ensure the patients’ safety and maintain hospital access during the pandemic.

Therefore, we set up a cohort of COVID-19 patients discharged home under nasal oxygen therapy with an electric-powered oxygen extractor and a pulse oximeter and remote monitoring by a telemedicine solution in collaboration with nurses and primary care physicians.

We aimed to evaluate the safety of this innovative home remote monitoring system. We also performed a systematic literature review on the home management of COVID-19 patients requiring oxygen and compared the different remote monitoring strategies and their effectiveness.

## 2 Methods

### 2.1 Recruitment

We performed a retrospective cohort study, including all consecutive COVID-19-confirmed patients with nasal oxygen therapy who consulted at our hospital (emergency department) and managed with remote monitoring (Terr-eSanté^©^), from October 2020 to April 2021. Patients could be included at hospital discharge or just after an emergency department (ED) consultation.

**Figure 1:**
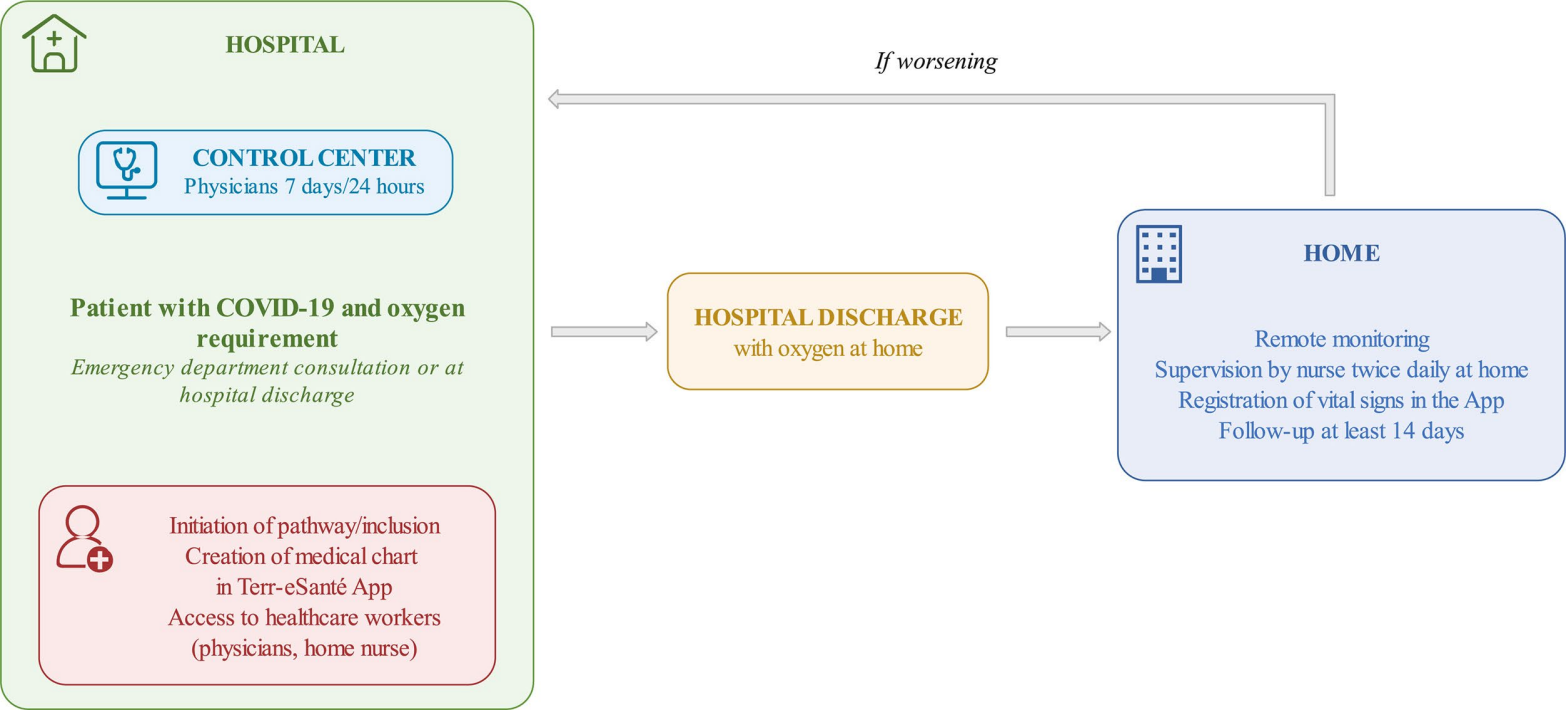
Patient pathway.

The web app (Terr-eSanté^©^) is an easy-to-use and free web application for healthcare workers (HCWs), which was designed to be straightforward and intuitive to use for patients and a panel of multidisciplinary healthcare professionals (infectious diseases specialists, emergency physicians, nurses, and primary care physicians). It allows several physicians (primary care, emergency, infectious disease, and other specialists) to exchange and communicate information on patients’ care and conditions (Figure 1).

Patients provided electronic consent for this remote monitoring program, but they also had access to the hospital medical ward outside of this web app.

To be included in the remote monitoring process, patients had to present all the following inclusion criteria after evaluation at our center (ED or in an acute medical ward): age ≥ 18 years old; oxygen flow requirement <5 L/min to obtain a saturation of at least 90%, temperature < 40°C, respiratory rate < 30c/min, arterial systolic pressure > 100 mmHg, and positive SARS-CoV-2 PCR results. Furthermore, the patients should not be isolated at home, should live within less than 1 h of our hospital, and should agree to participate in the process with nurse visits and compliance with remote monitoring. Finally, the patients should give their consent to be included in this cohort. Each patient could only be included once.

Exclusion criteria were: patient with already telemonitoring for any other medical cause, no oxygen requirement, and negative SARS-CoV-2 PCR results. Delay from symptoms onset and comorbidities was not an exclusion criterion.

Inclusion in the process of remote monitoring was performed by physicians of our hospital after consultation at the ED or at hospital discharge.

### 2.2 Remote monitoring process

The following data were registered by physicians: baseline characteristics, including age, gender, phone number or email address, date of first symptoms, the presence of cardiovascular disease, diabetes, chronic lung disease, and immunodeficiency (transplant, active cancer treatment, and uncontrolled HIV infection).

Treatment was prescribed according to guidelines (corticosteroids, prophylaxis of thrombosis, etc.). Then, patients were monitored at home by a nurse once or twice a day, according to their clinical status and severity, based on a medical prescription.

The monitoring period was at least 14 days but could be prolonged based on physician advice, especially if patients still required oxygen therapy.

During monitoring, in-home nurses collected the following data on the web app: blood pressure, temperature, oxygen saturation, and respiratory rate. In the web application, HCWs had access to the nurse’s transmissions, which were checked every day by the medical hospital COVID-19 team.

If the patient’s conditions worsened or if the nurse needed advice, an alert was created on the app or by phone call to the dedicated phone line of the medical hospital COVID-19 team.

During this period, the patients could have an in-site evaluation at our hospital on demand or after an alert. Consequently, patients could be hospitalized or discharged according to their conditions.

In case of rapid worsening, patients were advised to directly contact the national emergency number (Service d’Aide Médicale Urgente [SAMU]).

Registration, active monitoring, answers to alerts, coordination of care, and prescriptions were performed by a multidisciplinary medical team, with a coordinator nurse and a dedicated emergency department physician with a 24/7 service hotline for information between every HCW.

### 2.3 Statistical analysis

The primary endpoint was failure of remote monitoring, defined by unplanned hospitalization (excluding ED reevaluation) within the time of oxygen requirement.

Quantitative variables are presented as mean ± standard deviation (SD), or median and interquartile range (IQR). Qualitative variables are presented as the number of occurrences and relative frequencies.

We used χ2 tests to compare the distributions of categorical variables, whereas two-tailed, unpaired *t*-tests were used to compare the distributions of quantitative continuous variables. All reported *p*-values were based on two-sided tests, and a *p* < 0.05 was considered statistically significant.

To identify risk factors associated with failure, a univariate analysis by logistic regression was performed, using demographic and medical characteristics as well as all clinical and biological data. A multivariable analysis by logistic regression was then performed using all variables from the univariate analysis that had a *p* ≤ 0.05. The final model was obtained using backward stepwise regression with 0.10 thresholds.

Odds ratios (ORs) were calculated from the univariate and multivariable analyses to quantify association with failure during oxygen requirement with 95% confidence intervals (CIs). Analyses were performed with the use of the Statistical Package for Social Science (SPSS) version 26.0 (SPSS, Chicago, IL, United States).

The research was conducted in accordance with the Declaration of Helsinki and national and institutional standards. Patients were informed that their clinical data could be used, after anonymization, for research purposes. This study followed the Strengthening the Reporting of Observational Studies in Epidemiology (STROBE) reporting guidelines for cohort studies.

### 2.4 Literature review

Second, we performed a literature review of all articles on the literature review of studies on COVID-19 patients requiring oxygen with remote monitoring from 2020 to 2022.

We searched the Medline/PubMed and Web of Science databases with the following keywords: “remote monitoring,” “telemonitoring,” “COVID-19 monitoring,” and “oxygen requiring.”

## 3 Results

### 3.1 Screening

**Figure 2:**
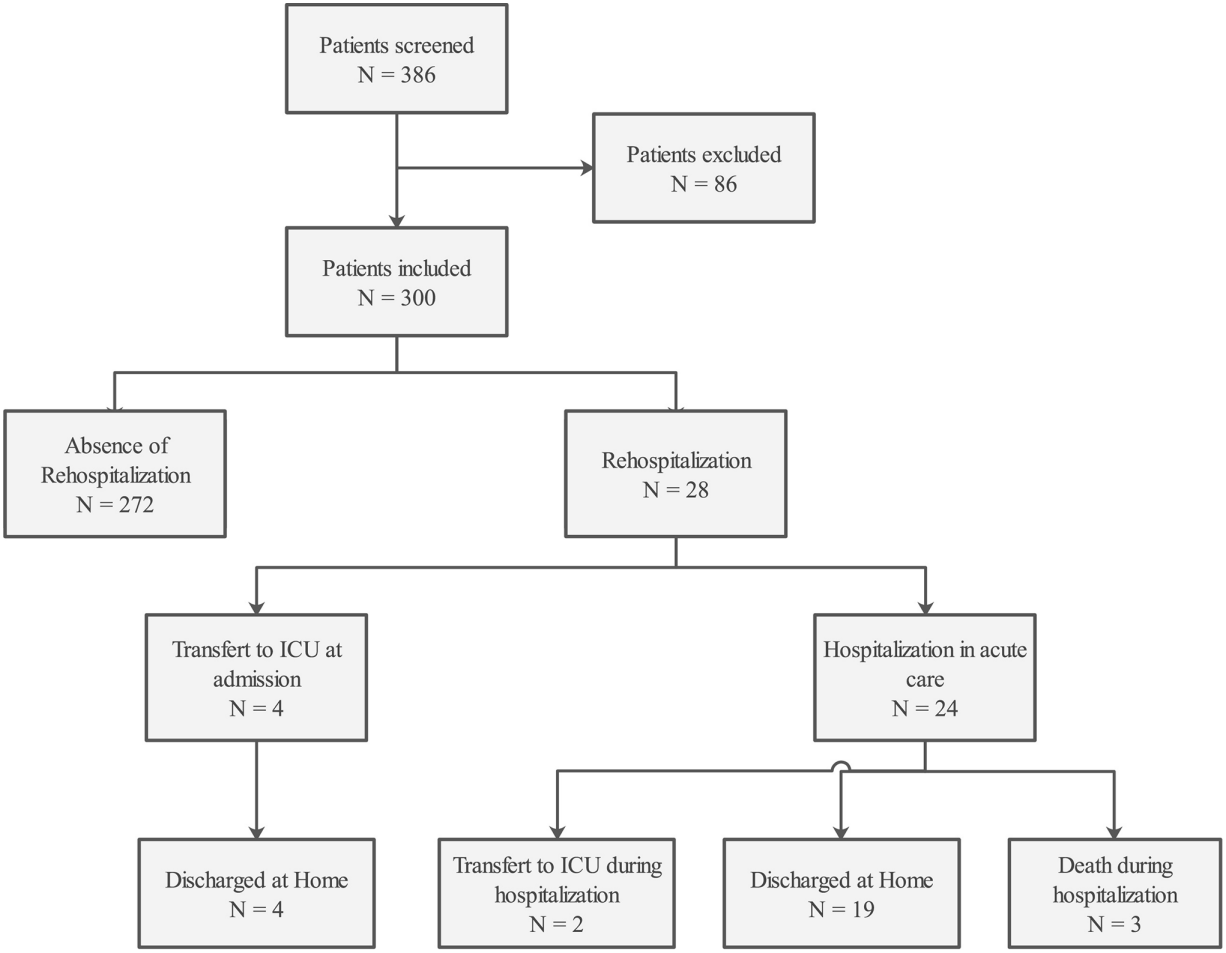
Flow chart and outcome of patients included in the home remote monitoring process.

During the study period, 386 COVID-19 patients were admitted to the ED or medical wards in our hospital. We excluded from our study 86 patients: 30 were already under monitoring at the beginning of the study, 20 were in remote monitoring for another disease (5 asthma, 1 pericarditis, 1 community-acquired pneumonia, 2 for pancreatitis, 1 for osteomyelitis, 1 for dehydration, 3 for acidocetosis, 1 for sigmoiditis, 1 for Horton disease, 1 for pulmonary embolism, 1 for severe dizziness, and 1 for appendicitis), 5 COVID-19 patients did not require oxygen, 21 medical charts were duplication, 6 patients were not able to benefit from remote monitoring, and 4 patients had an incomplete medical chart. The complete study flow chart is presented in Figure 2.

### 3.2 Patients

In our study, we analyzed a cohort of 300 distinct patients who had been discharged with oxygen therapy following COVID-19 pneumonia. Among these individuals, 72 (24.0%) were discharged from the emergency department, while 229 (76.0%) were released from inpatient admissions. The median age of the cohort was 51 years, with an interquartile range of 45 to 61 years, and of these patients, 184 (61.3%) were male patients. Detailed patient characteristics can be found in Table 1.

**Table 1:** Characteristics of COVID-19 patients included in the remote monitoring process.

	Total *N* = 300	Rehospitalization *N* = 28	No rehospitalization *N* = 272	*p*-value
Age (years, mean ± SD)	58.8 ± 14.1	64.5 ± 13.8	58.2 ± 14.1	0.024*
Male patients	184 (61.3)	19 (67.9)	165 (60.7)	0.457
**Comorbidities**				
Cardiovascular disease	117 (39.0)	15 (53.6)	102 (37.5)	0.097
Chronic respiratory failure	32 (10.7)	8 (28.6)	24 (8.8)	0.005*
Obesity	25 (8.3)	1 (3.6)	24 (8.8)	0.489
Diabetes mellitus	72 (24.0)	8 (28.6)	64 (23.5)	0.552
Immunosuppression	28 (9.3)	6 (21.4)	22 (8.1)	0.034*
Pulmonary embolism during initial hospital stay	10 (3.3)	0 (0)	10 (3.7)	0.607
**COVID-19 infection**				
Delay between onset of COVID-19 symptoms and start of telemonitoring (days, mean ± SD)	9.5 ± 4.4	5.6 ± 3.5	9.9 ± 4.3	<10^−5^*
Oxygen flow (L, mean ± SD)	2.0 ± 1.2	1.8 ± 1.4	2.0 ± 1.2	0.574
Length of hospital stay before start of telemonitoring (days, mean ± SD)	3.2 ± 2.9	3.9 ± 3.7	3.1 ± 2.8	0.190

* Statistically significant.

The median duration of follow-up for these patients was 26 days, with an interquartile range of 25 to 27 days. A total of 265 patients (88%) underwent successful oxygen weaning, with an average delay of 7.9 ± 7.3 days (ranging from 1 to 34 days) without any complications.

Notably, 28 out of the 300 patients (9.3%) required readmission to the hospital due to respiratory failure caused by COVID-19. Among these cases, six patients (2.0%) required intensive care unit (ICU) admission. Among the ICU admissions, four patients were directly admitted upon hospitalization, while two were transferred to the ICU on days 1 and 6 of their hospital stay. Of these readmissions, 26 were facilitated by nursing staff, while 2 patients sought consultation independently.

Within the subgroup of hospitalized patients, 19 out of the 28 (67.9%) who were readmitted for respiratory failure were eventually discharged home after successful oxygen weaning. Additionally, 36 out of the 300 (12.0%) medical charts indicated that monitoring was performed by a general practitioner.

Among the patients who were admitted to the ICU, five individuals required such intensive care. Their mean age was 60.2 ± 11.8 years. Among these patients, two out of five had a history of chronic respiratory failure, while one out of five was immunocompromised. Worsening in their condition occurred at an average of 11.4 ± 6.5 days.

Remarkably, the study documented a total of 3 out of 300 (1.0%) patient fatalities. None of these deaths occurred within the ICU, during the transportation back to acute care, or at home. Importantly, there were no instances of missed follow-up for any of the patients.

These unfortunate fatalities were observed in patients who had been admitted to acute care settings and were not transferred to the ICU due to underlying comorbidities. Specifically, one patient at the age of 48 with glioblastoma and a severe disability experienced respiratory failure on day 5. Another patient, aged 73, presented severe cardiovascular comorbidities (including stroke and ischemic cardiomyopathy) and experienced worsening on day 14. Finally, an 81-year-old patient with prostatic neoplasia deteriorated on day 13.

### 3.3 Univariable and multivariable analyses

We performed a univariable analysis to identify factors associated with unplanned rehospitalization. In this analysis, factors significantly associated with rehospitalization were age, chronic respiratory failure, delay from symptoms onset, and telemonitoring.

Thus, we performed a multivariate analysis using these parameters. In this analysis, factors associated with unplanned rehospitalization were immunosuppression (OR = 4.476), chronic respiratory failure (OR = 3.736), and a short delay between symptoms onset and the start of telemonitoring (OR = 1.344).

Results from the univariable and multivariable analyses are presented in Table 2.

**Table 2:** Univariable and multivariable analyses to identify factors associated with failure of remote monitoring, defined by unplanned hospitalization within the time of oxygen requirement.

	Univariable		Multivariable	
	OR (95%CI)	*p*-value	OR (95%CI)	*p*-value
Age (years)	1.034 (1.004–1.064)	0.026	1.027 (0.996–1.060)	0.087
Presence of chronic respiratory failure	4.133 (1.646–10.38)	0.003	4.476 (1.565–12.80)	0.005
Presence of immunosuppression	3.099 (1.137–8.445)	0.027	3.736 (1.208–11.552)	0.022
Delay between onset of COVID-19 symptoms and start of telemonitoring (days)	0.743 (0.657–0.842)	< 10^−5^	0.744 (0.653–0.847)	< 10^−5^

CI, confidence interval; OR, odds ratio.

## 4 Discussion

In this cohort study of 300 patients with COVID-19 pneumonia, discharged home with supplementary oxygen and remote monitoring, a low rate of unplanned hospitalizations, and no at-home deaths were reported.

Amidst the global COVID-19 pandemic, telemedicine has emerged as an essential tool with a global reach. Its role has been crucial in enhancing patient surveillance, containing disease spread, promptly identifying and managing the unwell, and most significantly, ensuring uninterrupted care for vulnerable individuals grappling with multiple chronic conditions.

The significance of telemedicine has been accentuated by the COVID-19 pandemic. It has facilitated vital communication between patients and healthcare providers, particularly when in-person visits were unfeasible or entailed infection risks or quarantine prerequisites (16). Teleconsultations have proven to be a secure and effective means for evaluating suspected COVID-19 cases, streamlining diagnosis and treatment processes while mitigating disease transmission risks (16). Moreover, telemedicine has enabled the uninterrupted provision of critical clinical services throughout the pandemic, particularly for high-risk scenarios and specific demographics (10, 11).

Although telemedicine has flourished during the COVID-19 era and gained traction in numerous nations, substantial gaps remain. Key challenges that demand attention for widespread telemedicine implementation include: (i) establishing comprehensive policies to govern telemedicine, license healthcare practitioners, safeguard patient confidentiality, and implement reimbursement structures; (ii) devising and disseminating pragmatic guidelines for the routine clinical utilization of telemedicine across diverse scenarios; (iii) enhancing the integration of telemedicine with conventional healthcare services; (iv) boosting awareness and willingness among healthcare professionals and patients to embrace telemedicine; and (v) surmounting disparities among nations and population subsets due to technological, infrastructural, and economic obstacles. Meeting these prerequisites in the near future could transform remote patient management into an indispensable tool for global healthcare systems, ultimately enhancing patient care and its quality (16).

Our results underline the safety of a remote monitoring oxygen program for patients with COVID-19 pneumonia, even among patients at an early stage of the disease and with several comorbidities, involving several HCWs: ED physicians, pulmonologists, infectious disease specialists, ICU physicians, nurses, and general practitioners.

Several experiences of remote monitoring for COVID-19 patients were described in the literature to prevent overwhelmed hospitals.

However, most of them included patients not requiring oxygen. For example, an experience of telemedicine with hotels playing the role of auxiliary hospitals was set up in Italy (13). It allowed us to discharge 258 mild COVID-19 patients to hotel rooms with nurses and physicians.

Another experience that involved primary care physicians was developed in Canada (14). Patients were monitored at home using daily questionnaires. If an alert occurred during follow-up, a video consultation or an in-person visit was performed.

The Covidon solution in the Greater Paris area is in line with the latter study (11). It provided home remote monitoring to more than one million patients with confirmed mild COVID-19 and was included by ED and primary care physicians. In case of worsening conditions, patients were referred to the ED or their general practitioner.

But these experiences provide mainly triage or home remote monitoring for mild COVID-19 in patients who do not require oxygen.

We included more severe patients with comorbidities and at high risk of severe disease. Moreover, most patients were included after consultation at the ED at the early stage of COVID-19, with possible worsening from days 7 to 10 from symptoms onset, due to a cytokine storm (17).

This strategy was acceptable only due to the high burden on hospitals and the rigorous process of remote monitoring. Factors associated with the failure of our remote home telemonitoring process were chronic respiratory failure, immunosuppression, and short delay between the onset of COVID-19 symptoms and the initiation of telemonitoring. Thus, these patients should be identified and cautiously monitored during their remote home telemonitoring.

Several other processes were set up during the COVID-19 crisis to manage remote monitoring of patients with oxygen requirements.

### 4.1 Literature review

We performed a literature review on COVID-19 patients requiring oxygen managed at home. We identified seven studies, including two in France, one in the US, two in the Netherlands, one in Türkiye, and one in India (18–24). The main study by Banerjee et al. was a large cohort study performed in the United States, which showed that patients discharged home with oxygen also had low rates of mortality and rehospitalization (18). In a French solution, the patients entered their own data via a web app, and no nurse was required (19). Moreover, a regional remote center was organized, and, depending on specific alerts, patients were contacted. Patients with oxygen were included at hospital discharge and required 3 L/min or less of oxygen therapy. The majority of patients were included after day 10 from symptoms onset and most of them experienced intensive care unit hospitalization prior to discharge under oxygen therapy. The duration of at-home oxygen therapy was longer.

In this present study, we also included patients at hospital discharge, but most of our patients were included straight after their consultation at the ED. They probably had fewer respiratory sequalae than patients included after ICU management, which could explain the short duration with oxygen therapy.

In our literature review, we identified seven other experiences of patients with COVID-19 requiring oxygen and managed at home (Table 3).

**Table 3:** Review of literature of COVID-19 patients with oxygen therapy and remote monitoring.

Author, journal, year (reference)	Study design	Process/pathway Nurse at home, digital app, auto vs. hetero surveillance	Inclusion criteria Early-stage vs. hospital discharge	Population	Outcome: unplanned hospitalization, mortality
Present study	Retrospective monocentric study	Web app and home nurse: Home remote monitoring with in-home nurse, connected via a web app with the emergency department	Early and late stage Requiring nasal oxygen therapy (≤ 5 L/min)	300 patients Male: 61.3% Age: mean 58.8 ± 14.1 years Immunosuppression: 8% Duration of oxygen therapy: mean 7.9 ± 7.3 days	Unplanned hospitalization: 9% Death: 3 patients
Dinh A. et al. Frontiers 2021 (19)	Retrospective multicenter study	Web app and phone call: Daily monitoring questionnaires on web app until oxygen therapy withdrawal In case of abnormal responses: alerts triggered in the regional control center, with monitoring by expert physicians	Late stage after hospitalization (after day 10) Requiring nasal oxygen therapy (≤ 3 L/min)	73 patients Male: 64.4% Age: median 62.0 years (IQR 52.5–69.0) Immunosuppression: 20.5% Duration of oxygen therapy: median 20 days (IQR 16–31)	No unplanned hospitalization No death
Banerjee J. et al. JAMA Open 2022 (18)	Retrospective bi-centric study	Nurse phone call: Nursing telephone follow-up, always with physician back-up	Early and late stage Requiring oxygen therapy (≤ 3 L/min) Stable without other indication for inpatient care	621 patients Male: 65.1% Age: median 51 years (IQR 45–61) Immunosuppression: 37.8% Duration of oxygen therapy: median 26 days (IQR 15–55)	Unplanned hospitalization: 8.5% All-cause mortality: 1.3% No death in the ambulatory setting
van Goor H. M. R. et al. J Clin Med 2021 (20)	Randomized, single-center, controlled trial	Web app and phone call by a medical student (intervention group): Questionnaire in the app 3 times daily with remote monitoring, and telephone contact daily for all patients by a group of trained medical students	Late stage Requiring oxygen therapy ≤3 L/min Patients identified by the treating physician (GP) Presence of supportive caretaker at home	31 patients (62 patients in total) Male: 54.8% Age: mean 55.1 ± 7.5 years Immunosuppression: not available Duration of oxygen therapy: mean 6.7 ± 7.5 days	Unplanned hospitalization: 6.5% No death No difference between two groups N.B.: visit to GP 2.4 times more visits in the control group
van Herwerden M. C. et al. Ned Tijdschr Geneeskd 2021 (21)	Retrospective monocentric study	Web app: Mobile App with remote monitoring (monitoring team unknown)	Requiring oxygen therapy ≤2 L/min Stable for 24 h	49 patients Population included unknown Duration of oxygen therapy: median 11 days	Unplanned hospitalization: 12.2%
Grutters L. A. et al. Eur Respir J 2021 (22)	Retrospective monocentric study	Phone call by the medical team: Twice daily control of oxygen saturation, temperature, and symptoms. Monitoring team: medical residents supervised by pulmonologists	Late discharge Requiring oxygen therapy ≤3 L/min After hospitalization with improving clinical trend	196 patients (320 patients in total) Male: 64% Age: mean 56 ± 12 years Immunosuppression: 3% Duration oxygen therapy: mean 11.7 ± 5.4 days	Unplanned hospitalization: 7% No death
Adly, A. S. et al. J Med Internet Res 2021 (23)	Randomized, single-blinded, controlled clinical trial	Web app and comprehensive supervision: Once daily videoconferencing by expert respiratory physiotherapists	Requiring oxygen therapy ≤5 L/min	60 Patients Including 30 patients receiving oxygen therapy (BiPAP ventilation), and 30 patients receiving physical therapy techniques and no oxygen Male: 33% Age: mean 32.2 ± 5.4 years Immunosuppression: not available Duration oxygen therapy: not available	Unplanned hospitalization: 6.6%
Sitammagari K. et al. Ann Intern Med 2020 (24)	Prospective monocentric cohort	Proactive home monitoring in a virtual acute care unit (VACU)	Requiring oxygen therapy ≤4 L/min	41 patients using supplemental oxygen 1 to 4 L/min Male: 44% Age: median 54 years (IQR 43–64)	Unplanned hospitalization: 13% Hospitalization in ICU: 42% No death

GP, general practitioner; ICU, intensive care unit; IQR, interquartile range.

Included patients could be at an early or late stage of the disease, with a median age comprised between 51 and 62 years, with a majority of male patients. Patients at high risk were included (37.8 to 67%). Home monitoring was performed via a web app in five of seven studies, phone calls in three of seven studies, and web apps and phone calls were associated in two studies. The monitoring team included nurses in one study, a medical team in four of seven studies, and medical students in one study. Finally, the mortality rate was low (maximum 1.93%), as was unplanned hospitalization (max 12%).

### 4.2 Limitations

Our study has several limitations. First, it is an observational study with potential bias, considering the indication and selection of patients. Moreover, no control group is available.

Second, the study population is heterogeneous: oxygen therapy at the early stage of the disease vs. oxygen therapy at discharge (late stage of the disease). Moreover, the healthcare systems and organizations differ between countries. All of these factors limit the generalizability of these study findings.

Finally, we could question the best primary endpoint for these types of process evaluations. As the primary goal is to ease overburdened hospitals, the best criteria seem, in our opinion, to be unplanned hospitalization, especially in the ICU, independent of patients’ mortality rates. Thus, our process seems promising for relieving hospitals overwhelmed with COVID-19 patients requiring oxygen.

## 5 Conclusion

In this cohort study, ambulatory management of patients with COVID-19 pneumonia requiring home oxygen through a remote monitoring process was associated with a low unplanned hospitalization rate, especially in the ICU. This management strategy may be considered for patients with COVID-19 pneumonia. Moreover, similar results have been reported worldwide with different types of solutions, reinforcing the possible management at home of COVID-19 patients with oxygen therapy.

## Data availability statement

The raw data supporting the conclusions of this article will be made available by the authors, without undue reservation.

## Ethics statement

The requirement of ethical approval was waived because of the study’s retrospective nature on medical charts data. The study was conducted in accordance with the local legislation and institutional requirements. The ethics committee/institutional review board also waived the requirement of written informed consent for participation from the participants or the participants’ legal guardians/next of kin. Patients were informed that their clinical data could be used, after anonymization, for research purposes.

## Author contributions

JC: Data curation, Formal analysis, Writing – original draft. AD: Conceptualization, Formal analysis, Investigation, Supervision, Validation, Writing – original draft. ED’A: Writing – review & editing. FB: Formal analysis, Writing – original draft. KJ: Writing – review & editing. CD: Formal Analysis, Investigation, Writing – original draft. CG: Conceptualization, Supervision, Writing – original draft.
